# Morning and night symptoms in primary care COPD patients: a cross-sectional and longitudinal study. An UNLOCK study from the IPCRG

**DOI:** 10.1038/npjpcrm.2016.40

**Published:** 2016-07-21

**Authors:** Ioanna Tsiligianni, Esther Metting, Thys van der Molen, Niels Chavannes, Janwillem Kocks

**Affiliations:** 1 Department of General Practice, University Medical Center Groningen, University of Groningen, Groningen, The Netherlands; 2 Groningen Research Institute for Asthma and COPD, University Medical Center Groningen, University of Groningen, Groningen, The Netherlands; 3 Department of Public Health and Primary Care LUMC, Leiden, The Netherlands

## Abstract

COPD symptoms show a diurnal variability. However, morning and night variability has generally not been taken into consideration in disease management plans. The aims of this study were to cross-sectionally assess morning and night symptom prevalence and correlation with health status and disease severity in COPD, and to determine to what extent they could predict longitudinal outcomes, exacerbations and health status. A further aim is to explore whether the CCQ is able to depict this morning/night symptomatology. We included 2,269 primary care COPD patients (58% male, 49% current smokers, with a mean age of 65±11 years) from a Dutch Asthma/COPD service. Spirometry, patient history, the Clinical COPD Questionnaire(CCQ) and the Asthma Control Questionnaire(ACQ) were assessed; we used the latter to evaluate morning (question 2) and night symptoms (question 1). A total of 1159 (51.9%) patients reported morning symptoms (ACQ question 2>0) and 879 (39.4%) had night complaints (ACQ question 1>0). Patients with morning/night symptoms were mostly smokers and had on average poorer lung function, higher CCQ scores and used more rescue inhalers (*P*<0.0001). Patients using long-acting muscarinic antagonists (LAMAs) had less night symptoms, showing a possible favourable effect. Only a small proportion of stable or slightly unstable patients (CCQ total scores <2) had severe morning symptoms (ACQ 2⩾4: *n*=19, 1.1%) or severe night symptoms (ACQ 1⩾4: *n*=11, 0.7%). Night symptoms seemed to predict future exacerbations; however, baseline exacerbations were the strongest predictors (*n*=346, OR:4.13, CI: 2.45−6.95, *P*<0.000). Morning symptoms increased the odds of poor health status at follow-up (*n*=346, OR:12.22, CI:4.76−31.39, *P*<0.000). Morning and night symptoms in COPD patients are common, and they are associated with poor health status and predicted future exacerbations. Our study showed that patients with morning/night symptoms have higher scores in CCQ, and therefore we do not really miss patients with high morning/night symptomatology when we only measure CCQ. Severe morning symptoms predicted worsening of COPD health status.

## Introduction

Chronic obstructive pulmonary disease is a progressive disease characterised by persistent airflow limitation associated with substantial morbidity and mortality.^1^ The primary symptoms of the disease are dyspnoea, cough and sputum production.^1^ Starting in 2011, until the latest revision of GOLD 2015, pulmonary function, symptoms/health status assessment (as assessed by mMRC, CAT or CCQ)^2–4^ and number of exacerbations are included in the COPD algorithm for patient classification and management.^1^

Recognising the importance of the severity of symptoms, patients are categorised in the A,C (few symptoms) or B,D (more symptoms) categories.^1^ Next, a measure of risk is included in the algorithm. Both low lung function (forced expiratory volume in 1 s (FEV_1_) below 50% of predicted) or previous exacerbations (more than 2 in the last year or a hospital admission) result in classification in high risk (C,D).^1^ The classification in A–D will lead to management suggestions in the GOLD guideline.

Consequently, the decision tree for management is based on this classification. With this categorisation, GOLD recommendations attempt a more personalised approach to disease management.^1^ Symptom assessment is included in the three questionnaires used by GOLD (CAT, mMRC and CCQ) and in most patient-reported outcome questionnaires. However, none of them include a specific question for diurnal symptom variability, morning or night.^1–5^ CAT has a question on sleep, but it only assess whether the patient sleeps soundly or not.^3^ GOLD guidelines do not mention morning and night-time symptoms as targets for therapeutic interventions, and they do not offer a specific guidance on appropriate management strategies or pharmacological interventions for patients with COPD who report diurnal variability in their symptoms.^1,6^ Moreover, it appears that patients do not report symptom variability and do not modify treatment when symptoms worsen,^6,7^ and thus physicians are unlikely to discuss diurnal variability with patients.^6,8^

Not only asthma but also COPD shows diurnal variability in physiological spirometric parameters of lung function and peak expiratory flow (PEF).^9,10^ Recent studies have shown that COPD symptoms follow physiological diurnal variability and vary over time,^7,11,12,13^ as well as by geographical areas.^7^ Morning^7,11–18^ and night symptoms^6,7,13,19–23^ are prevalent and burdensome for patients with COPD, compromising patients’ ability to perform tasks throughout the day.^7,8,11–15,24^ It seems that patients experience the biggest increase in respiratory symptoms during the early hours of the morning, followed by another increase in the night time,^11,13,16^ especially in patients with severe COPD.^13,15^ This coincides with the circadian variation in lung function. In particular, morning symptoms have been found to be associated with worse health status,^13,18^ sleep quality,^13^ higher anxiety and depression,^13^ and more exacerbations.^7,18^ In the same way, night symptoms are associated with worse health status^13,19^ and seem to be able to predict future exacerbations.^20^ However, most of the studies concerning the variability of symptoms have only assessed morning or night symptoms in specific groups of COPD patients.

Therefore, the aims of our study were to explore the prevalence of morning and night symptoms, their distribution in different GOLD stages and grades and their correlation with lung function and health status, as well as to longitudinally explore their role in predicting future events such as worsening of health status and exacerbations. The null hypothesis was that morning and/or night symptoms were a distinct phenotype of highly symptomatic patients not captured by CCQ.

We conducted this study in a real-life setting with the aim of having a high external validity, as a primary care population has been used. Moreover, we aimed at assessing both morning and night symptoms rarely assessed simultaneously, as previously mentioned, in other studies.

## Results

### Descriptive analyses

In our COPD population (*n*=2269), 1,159 (51.9%) and 879 (39.4%) patients reported morning symptoms and night symptoms, respectively. Sociodemographic and baseline characteristics of the COPD patients are depicted in Table 1. A subset of patients had severe morning and night symptoms, with *n*=109 (4.9%) and *n*=74 (3.3%), respectively (Table 1). Only a small proportion of patients with stable or slightly unstable COPD (CCQ total scores<2) had severe morning symptoms (ACQ 2⩾4: *n*=19, 1.1%) or severe night symptoms (ACQ 1⩾4: *n*=11, 0.7%; Table 1). The correlation between morning and night symptoms was moderate (*r*=0.53), as was also their correlation with the CCQ (*r*=0.58, *r*=0.52, respectively). More details on correlations are depicted in Table 2.

### Inferential analyses: cross-sectional differences

**Figure 1:**
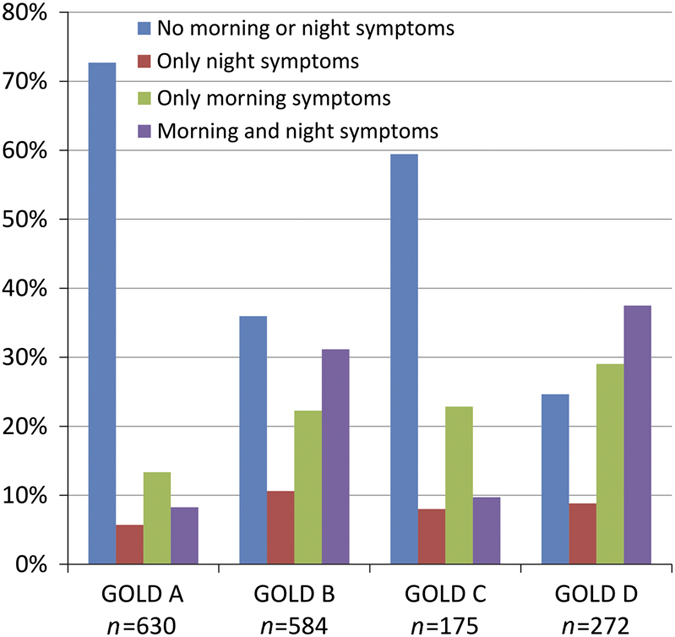
Percentages of patients with no morning and night symptoms, only night or morning symptoms and patients with both morning and night symptoms within the GOLD A,B,C,D categories.

**Figure 2:**
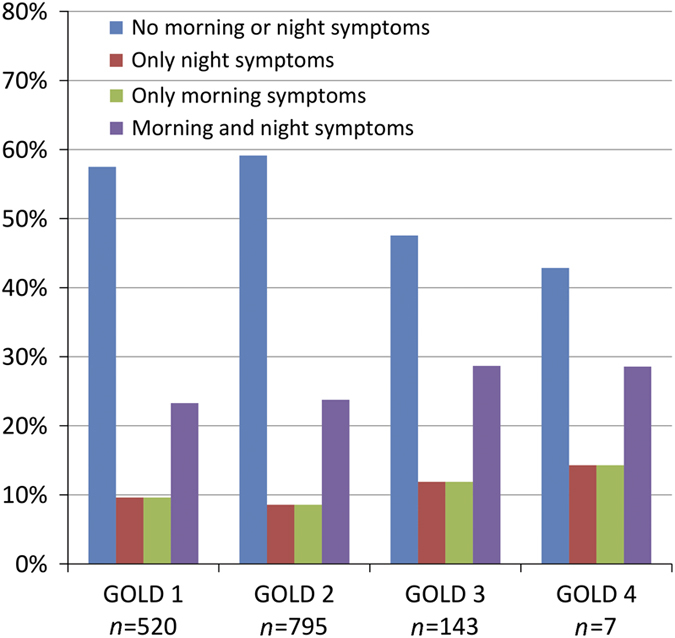
Percentages of patients with no morning and night symptoms, only night or morning symptoms and patients with both morning and night symptoms within the GOLD 1,2,3,4 categories.

Patients with morning/night symptoms had on average poorer lung function, higher CCQ scores, higher disease severity, were mostly smokers and used more rescue inhalers and less Long-Acting Muscarinic Antagonists (LAMA) compared with patients without morning/night symptoms (Table 3). *Post hoc* tests showed that patients with only night symptoms used LAMA less frequently (binominal regression OR =0.50 (0.31–0.80), *P* value =0.004). We did not find similar effects in other types of medication. The prevalence of GOLD D in patients without morning or night symptoms was 8%, whereas 28.9% of the patients with morning and night symptoms were GOLD D patients (Figure 1). This pattern was not found in the GOLD 1–4 grades (Table 3, Figure 2), which might indicate that FEV_1_ is not related to morning and night symptoms.

### Inferential analyses: longitudinal differences

Baseline exacerbations were the strongest predictors of exacerbations at follow-up, whereas patients with severe morning and moderate night symptoms had a higher risk of having an exacerbation compared with patients without these symptoms (Table 4). Severe morning symptoms were strong predictors of poor health status. This relationship was even stronger than the predictive value of baseline health status on health status at follow-up. Morning or night symptoms did not predict FEV_1_% predicted at follow-up (Table 4).

## Discussion

### Main findings

Our study showed that morning and night symptoms were common in real-life primary care COPD patients. Patients with morning/night symptoms had on average poorer lung function, higher CCQ scores and most of them were smokers. Only a small proportion of patients with stable or slightly unstable COPD (CCQ total scores <2) had severe morning symptoms (ACQ 2⩾4: *n*=19, 1.1%) or severe night symptoms (ACQ 1⩾4: *n*=11, 0.7%), rejecting our null hypothesis that morning and/or night symptoms were a distinct phenotype of highly symptomatic patients not captured by CCQ.

This is extremely important, as it shows that patients with morning/night symptoms do not represent a distinct phenotype. Morning/night symptoms predicted the number of exacerbations in the following 10–17 months, but the effect disappeared after adding baseline exacerbations in the model. Severe morning symptoms were more strongly predictive of health status than night symptoms at follow-up. Morning or night symptoms did not predict FEV_1_ decline within a year.

### Interpretation of findings in relation to previously published work

#### Morning/night symptoms: prevalence and correlations

Our study showed that morning (51.9%) and night symptoms (39%) were common in COPD patients (Tables 1 and 3) in accordance with several studies that showed that symptoms are worse in the morning.^7,8,11,12,13,15,18^ However, studies show a great heterogeneity in results because of differences in COPD populations, the study design, the ways to measure these symptoms and their severity. Therefore, some studies showed lower prevalence than our study in morning symptoms (37%–46% depending on GOLD severity^12,18^), whereas other studies have shown higher prevalence than our study,^7,13,15^ with prevalences that reached higher than 65% for some symptoms such as morning breathlessness.^7,12,13^ In our study, night symptom prevalence reached the 39% of patients with COPD. Partridge *et al.*^12^ reported night-time symptoms to be around 25–34% depending on severity, whereas a higher rate reaching 68% was reported in another study.^19^ In the study by Lange *et al.*, in which a very large cohort of COPD patients (*n*=6,616) was examined, the prevalence of night-time dyspnoea was 4%, increasing to 9% and 16% in severe and very severe patients, respectively.^20^

In our study, 32.04% of patients had both morning and night symptoms, confirming the findings of the ASSESS study, which showed that only 10.6% of patients had symptoms in only one part of the day, whereas the majority had both morning and night symptoms.^13^ Correlations of morning and night symptoms between them was modest (*r*=0.53), suggesting that they may measure a different concept.

#### Morning/night symptoms: gender and smoking

We did not find gender differences regarding morning/night symptoms, which is in concordance with the study from Roche *et al*.^18^

The proportion of smokers was higher in patients with morning and/or night symptoms. Patients experiencing morning symptoms were more likely to be current smokers as in the study by Roche *et al*.^18^ In our study, the pattern was completely the opposite in patients who quit smoking at least 12 months earlier, as the majority of them no longer reported morning and night symptoms, confirming the benefits of smoking cessation.

#### Morning/night symptoms and lung function

Patients with morning or/and night symptoms had on average poorer lung function (FEV_1_, FEV_1_/FVC). In the same way in the Roche *et al* study, patients with morning symptoms had worse lung function.^18^ In our study, the correlation between both morning and night symptoms with lung function (Table 2) was low, confirming that symptoms in general do not correlate well with lung function, which is already known.^1^ Moreover, our longitudinal analysis showed that morning symptoms or night symptoms did not predict FEV_1_ decline in 1 year (Table 4).

#### Morning/night symptoms and disease severity

Differences existed between the four categories (patients with both morning and night symptoms, any or none of these symptoms) within the different GOLD stages (I, II, III and IV) and A,B,C,D categories (Table 3, Figures 1 and 2). However, only a low number of patients with low symptomatology according to CCQ had high morning symptoms/night symptoms (1.5% and 0.7%, respectively), demonstrating that CCQ does capture the morning/night symptomatology well. Two studies have shown a significant association between night-time symptoms and the severity of airflow obstruction in patients with COPD;^19,20^ however, our study was in accordance with the study of Miravittles *et al*.,^13^ showing that the prevalence of night-time symptoms was comparable across all severities of airflow limitation. These data suggest that the presence of night-time symptoms is not merely a consequence with severe airflow limitation.

The pattern of morning and/or night symptoms was better represented in the A,B,C,D categories (Figure 1) than in GOLD 1,2,3,4 (Figure 2). In an international study, patients in category D followed by the category B showed the highest rates of night symptoms, which was similar to our study.^19^ Our study, however, took the next step by also showing that morning and morning plus night symptoms were more prevalent in category D. This in a sense is expected, as categories B and D are the categories with more symptomatology.^1^ However, patients in categories A and C (low symptoms categories) also had a high rate of morning and/or night symptoms, similar to another study on night symptoms.^19^ Similar findings were also observed in the study by Lange *et al.*, which showed a high rate of night-time dyspnoea in descending rates in D, B, C and A.^20^

#### Morning/night symptoms and health status

Patients experiencing morning and/or night symptoms had worse health status, as assessed by CCQ. In addition, in other studies, a worse health status was found when morning symptoms were present, as assessed either by CAT^13,18^ or EQ-5D.^18^ In the same way, a worse health status was found when night symptoms were present, as assessed either by CAT^13^ or EQ-5D.^19^ In addition, patients with COPD and poor sleep quality^25^ or insomnia^26^ had mentioned a worse quality of life.

A modest correlation between morning and night symptoms and CCQ was found, suggesting that they may measure a different concept and should therefore be taken into consideration when tailoring treatment and interventions. However, morning symptoms had a greater correlation and explained in a greater degree the variance in health status than night symptoms. In addition, only a small proportion of patients with stable or slightly unstable COPD (CCQ total scores <2) had severe morning/night symptoms.

Severe morning symptoms and baseline CCQ scores compared with FEV_1_ were the strongest predictors of CCQ scores at follow-up. As health status is included in the COPD GOLD management algorithm, it would be rational to suggest to clinicians to also control morning symptoms to avoid worsening of health status.

#### Morning/night symptoms and exacerbations

In our study, patients with morning and night symptoms, as well as those with only morning symptoms, had more exacerbations. COPD symptoms first thing in the morning have been shown to be independently associated with more exacerbations.^7,18^

Our study showed that baseline exacerbations were the strongest predictors of future exacerbations compared with morning/night symptoms. It is well known that previous exacerbations may be considered as a different phenotype showing susceptibility to exacerbations.^27,28^ The ECLIPSE study showed that the major determinant of frequent exacerbations in all GOLD stages of COPD severity was a history of exacerbations.^28^ Lange *et al.* have shown that nocturnal symptomatic COPD patients had a 2.3-fold higher risk of suffering a future exacerbation; however, this study assessed only night-time symptoms, contrary to our study that showed that morning symptoms were the symptoms that predicted mostly future exacerbations as compared with night symptoms.^20^ Omachi *et al.*^22^ also showed that sleep disturbances predict future exacerbations, mortality and emergency utilisation.

#### Morning–night symptoms and medication use

In our study, significant differences were observed in short-acting bronchodilators and LAMA use with morning and night symptoms/only morning/only night/no morning–night symptoms (in a descending order) using more SABA. The fact that these patients used more SABA shows that despite optimum maintenance therapy those patients still have a need for symptom relief. Similarly, in the study by Kessler *et al* , patients with day-time variability used more emergency inhalers (35.7%).^7^ In the study by Roche *et al*, there was an increased use of maintenance (LABA, LAMA) and combination therapies when morning symptoms were present.^18^ Because of the recent introduction of fixed LABA/LAMA combination, no patients used a fixed LABA/LAMA combination in one device at the time of analysis. In our study, no differences in LABA and combination therapies were found on morning/night symptoms. However, there were great differences in morning/night symptoms between LAMA users and those who did not use LABA, showing a possible protective effect. In the same way, in the study by Kim *et al*, LAMAs (odds ratio [OR], 6.971; 95% confidence interval, 1.317 to 11.905 *P*<0.0143) were identified as significantly related to the absence of morning symptoms.^15^ The *post hoc* analysis in our study, however, showed that patients using LAMA had less night symptoms, whereas LAMA was not effective in the patients with morning symptoms. Therefore, this group of patients with only night symptoms may benefit from the use of LAMA in the future.

### Strengths and limitations of this study

We designed this real-life study using patients from a primary care population in order to ensure a high external validity. To our knowledge, it is also the first study that assessed longitudinally the role of both morning and night symptoms in predicting exacerbations and health status. This study enroled patients independent of disease severity, from the usual consultations in primary care, and therefore these findings may be applicable to a wider population. However, as a cross-sectional study, there is a lack of ability to show cause and effect. The longitudinal analysis provides valuable additional information.

Our study used the ACQ questions to assess morning and night symptoms. This may be considered invalid because the ACQ is not designed nor validated for the use in COPD patients. However, no validated instrument exists that assesses both morning and night symptoms in COPD patients, and all patients completed both questionnaires in the study at baseline, as the diagnosis had not yet been established. Up until now, studies conducted to assess those symptoms used different tools, questionnaires,^20^ patient record forms,^7,18^ the clinical symptom questionnaire,^15^ interviews^7,11,12^ or the a ‘Night-time/Morning and Day-time Symptoms of COPD questionnaire’.^13^ Questions similar to the ACQ questions have been used in the majority of these studies to assess morning and night symptoms. Some examples are shown in the study by Kim *et al*.^15^ ‘When were your symptoms the most troublesome for you? On waking / In the morning / In the afternoon / In the evening / At night’ and in the study by Lange *et al.*^20^ ‘do you sometimes wake up in the night because of breathlessness/difficulties with breathing’? Nevertheless, we believe that these possible limitations do not invalidate the results obtained.

### Implications for future research, policy and practice

Symptom variability may be an additional sign that the disease is not under control. Patients suffering more from morning and night symptoms are at a higher risk of future exacerbations. A new patient-reported outcome instrument, the Night-time Symptoms of COPD Instrument (NiSCI), has been recently developed.^29^ However, unfortunately, to date no validated questionnaires that measure both morning and night symptoms are available.

Data suggest that only a minority of patients adapt their treatment in response to worsening of symptoms^7^ and that patients are taking medications too late in the day to benefit fully from their potential effects.^12,30^ Further work is now warranted to identify the periods in which the symptoms are more intense and the medications that improve these morning or night symptoms. Recently, in a meeting focusing on the night-time symptoms and sleep disturbances of COPD patients, a panel of experts highlighted the lack of interest in clinical studies on these aspects and suggested a call for action.^6^

### Conclusions

Morning and night symptoms are common in COPD patients. Patients with morning/night symptoms have on average poorer lung function, higher CCQ scores, higher severity, are mostly smokers and use more rescue inhalers; these findings are not reflected in the current GOLD approach. Our study shows that very few patients experience many morning or night symptoms when the CCQ scores are low, suggesting that having morning/night symptoms does not reflect a specific phenotype. Patients using LAMA have less night symptoms, showing a possible favourable effect. Severe morning symptoms increased the odds of poor health status at follow-up. Morning and night symptoms seemed to predict future exacerbations; however, baseline exacerbations are the strongest predictors.

## Materials and Methods

### Participants setting

We included the data from 2,269 COPD patients (57.8% male, 49.0% current smokers, with a mean age of 65.3±10.8 years) between 2007 and 2013, assessed by a primary care Asthma/COPD service^31^ that operates in the Netherlands. In this service, a pulmonologist reviews patients for general practitioners after assessment by the primary care laboratory. All patients undergo a systematic assessment. The general practitioners referred patients with respiratory complaints to the service (GP) for diagnostic assessment. Pulmonologist diagnose with COPD, asthma, asthma/COPD overlap, restrictive disease or diagnosis uncertain. We have only included ‘pure’ COPD without overlap or a history of asthma in this study.

In this study, we used anonymous assessment data. According to Dutch regulations, a separate ethics committee approval is not required, because routinely collected health-care data are used after anonymisation.

### Spirometry-patients reported outcomes

Spirometry was performed including reversibility testing at baseline. All patients’ reported outcomes were part of the regular assessment procedure of the AC service. Patients, regardless of their current diagnosis, filled in a history questionnaire containing questions about medication use, exacerbations, age of onset, allergies, hyper-reactivity and family history. In addition, the history questionnaire included the Asthma Control Questionnaire (ACQ)^32^ and the Clinical COPD Questionnaire (CCQ).^4^ Therefore, our COPD patients had ACQ scores available.^32^ For the assessment of morning and night symptoms, the ACQ question 2 (‘On average, during the past week, how bad were your asthma symptoms when you woke up in the morning?’) and question 1 (‘On average, during the past week, how often were you woken by your asthma during the night?’) were used.

### Statistical analyses

IBM SPSS version 22 was used to analyse the data. Missing data were scarce (<1%) and assumed to be missing at random and therefore not imputed.

### Descriptive analysis

We have described the prevalence of morning and night symptoms in our COPD population along with a thorough description of our population in terms of age, gender, age of onset, smoking status, severity of COPD, symptoms, health status and medication use. We calculated correlations between morning/ night symptoms and age, age of onset, CCQ, exacerbations, wheezing and lung function. We categorised the ACQ question 1 scores and ACQ question 2 scores into ‘No symptoms (ACQ=0),’ ‘Mild symptoms (ACQ=1–3)’ and ‘Highly symptomatic (ACQ⩾4).’

### Inferential analyses: cross-sectional differences

We based the cross-sectional analyses on baseline data from 2,369 patients. We tested cross-sectional baseline differences in patient characteristics, COPD severity and symptoms between patients with morning symptoms or/and night symptoms and patients without morning or night symptoms with a t-test or Mann–Whitney *U*-test when we had continuous variables. We used the *χ*^2^-test if the variable was discrete and *post hoc* binomial regression to compare LAMA use in patients without morning/night symptoms with patients in the other groups.

### Inferential analyses: Longitudinal differences

Before GPs referred to the AC service, they indicated whether they wanted to follow-up the patients themselves or whether patients should be scheduled for yearly follow-up assessments by the AC service. We included data from patients scheduled for the yearly follow-up assessment by the AC service. We assessed these patients in 10 to 17 months after their baseline visit (*n*=445) and compared baseline results with follow-up data. We also used multinomial logistic regression to examine whether morning symptoms or night symptoms could predict the risk of an exacerbation or poor health status (CCQ total score ⩾1, according to the GOLD guidelines).^1^ We took into account, respectively, baseline exacerbation or baseline health status. Through linear regression analysis, we assessed the effect of morning/night symptoms on FEV_1_ decline.

## Figures and Tables

**Table 1:** Sociodemographic and baseline characteristics of the COPD patients

*Variable*	n* total*	*Statistic*	
*Gender and age*			
Men	2,269	*n* (%)	1,312 (57.8)
Age	2,269	Mean (s.d.)	65.3 (10.8)
Age of onset	1,992	Mean (s.d.)	52.4 (19.7)
Exacerbations	2,263	Mean (s.d.)	0.72 (1.1)
			
*Lung function post bronchodilator*			
FEV_1_ (l)	2,262	Mean (s.d.)	2.0 (0.7)
FEV_1_% predicted	2,263	Mean (s.d.)	69.3 (18.0)
FVC (l)	2,210	Mean (s.d.)	3.5 (1.0)
FVC % predicted	2,211	Mean (s.d.)	98.8 (18.4)
FEV_1_ / FVC	2,260	Mean (s.d.)	56.1 (11.0)
Reversibility	2,063	Mean (s.d.)	6.2 (8.0)
			
*Smoking history (age⩾18 years)*	*2,261*		
Never smoked		*n* (%)	80 (3.5)
Ex-smoker		*n* (%)	1,069 (47.1)
Current smoker		*n* (%)	1,112 (49.0)
Smoking history missing		*n* (%)	8 (0.4)
			
*GOLD stage*			
GOLD 1 (FEV_1_>80%)	2,269	*n* (%)	670 (29.5)
GOLD 2 (50%<FEV_1_<80%)		*n* (%)	1,257 (55.4)
GOLD 3 (30%<FEV_1_<50%)		*n* (%)	317 (14.0)
GOLD 4 (FEV_1_<30%)		*n* (%)	25 (1.1)
GOLD A	2,264	*n* (%)	646 (28.5)
GOLD B		*n* (%)	916 (40.4)
GOLD C		*n* (%)	176 (7.8)
GOLD D		*n* (%)	526 (23.2)
GOLD ABCD missing		*n* (%)	5 (0.2)
			
*Patient-reported outcomes*			
ACQ question 2: morning symptoms	2,231	Mean (s.d.)	1.1 (1.3)
Symptomatic (ACQ 2⩾1)		*n* (%)	1,159 (51.9)
Severe symptoms (ACQ 2⩾4)		*n* (%)	109 (4.9)
*Of which* stable or slightly unstable COPD patients (CCQ<2)	109	*n* (%)	19 (1.1)
ACQ question 1: night symptoms	2,233	Mean (s.d.)	0.7 (1.1)
Symptomatic (ACQ 1⩾1)		*n* (%)	879 (39.4)
Severe symptoms (ACQ 1⩾4)		*n* (%)	74 (3.3)
*Of which* stable or slightly unstable COPD patients (CCQ<2)	74	*n* (%)	11 (0.7)
Clinical COPD questionnaire total score (CCQ)	2,263	Mean (s.d.)	1.4 (1.0)
Stable (CCQ<1)		*n* (%)	817 (36.0)
Not entirely stable (CCQ⩾1 and<2)		*n* (%)	869 (38.3)
Unstable (CCQ⩾2 and <3)		*n* (%)	391 (17.2)
Very unstable (CCQ⩾3)		*n* (%)	177 (7.8)
CCQ missing		*n* (%)	15 (0.7)
Asthma Control questionnaire total score	2,224	Mean (s.d.)	
Controlled (ACQ <0.75)		*n* (%)	848 (37.4)
Partially controlled (ACQ⩾0.75 and <1.50)		*n* (%)	625 (27.5)
Uncontrolled (ACQ⩾1.50)		*n* (%)	753 (33.2)
ACQ missing		*n* (%)	43 (1.9)

Abbreviations: ACQ, Asthma Control Questionnaire; CCQ, COPD Clinical Questionnaire; COPD, chronic obstructive pulmonary disease; FEV_1_, forced expiratory volume in 1 s; FVC, forced vital capacity; GOLD, Global initiative for chronic obstructive disease.

**Table 2:** Correlations between symptoms and patient characteristics

	n	*Morning symptoms* a		*Night symptoms* b	
		r	*Proportion explained variance (*r^*2*^)	r	*Proportion explained variance (*r^*2*^)
*Characteristics*					
Age	2,233	−0.12	1%	−0.1	1%
Age of onset	1,992	−0.05	0%	−0.02	0%
FEV_1_ predicted	2,227	−0.16	3%	−0.1	1%
FEV_1_/FVC predicted	2,224	−0.11	1%	−0.03	0%
Mean number of exacerbations last year	2,228	0.13	2%	0.12	1%
					
*Symptoms*					
Night symptoms (ACQ question 1)	2,233	0.53	28%	1	100%
Morning symptoms (ACQ question 2)	2,231	1	100%	0.53	28%
Sputum production (CCQ question 6)	2,231	0.46	21%	0.39	15%
Wheezing (ACQ question 5)	2,232	0.45	20%	0.44	19%
					
*Health status*					
CCQ subscale functional status	2,233	0.44	19%	0.39	15%
CCQ subscale mental status	2,230	0.41	17%	0.39	15%
CCQ subscale symptoms	2,232	0.59	35%	0.52	27%
CCQ total scale	2,229	0.58	34%	0.52	27%

Abbreviations: ACQ, Asthma Control Questionnaire; CCQ, COPD Clinical Questionnaire; COPD, chronic obstructive pulmonary disease; FEV_1_, forced expiratory volume in 1 s; FVC, forced vital capacity.

a ACQ question 2.

b ACQ question 1.

**Table 3:** Differences in characteristics between patients with no morning and night symptoms, only morning or night symptoms and patients with both morning and night symptoms

		*No morning/night symptoms*	*Only morning symptoms, no night symptoms*	*Only night symptoms, no morning symptoms*	*Morning and night symptoms*	P *value*
		n*= 908*	n*=444*	n*=164*	n*=715*	
*Characteristics*						
Age	Mean (s.d.)	65.9 (0.4)	63.2 (0.6)	64.8 (0.9)	65.4 (0.6)	<0.000
Age of onset	Mean (s.d.)	52.9 (0.8)	50.1 (1.2)	54.9 (1.4)	53.8 (1.1)	NS
Male	*n* (%)	495 (58.9)	188 (56.5)	74 (54.4)	203 (57.5)	NS
Exacerbations	Mean (s.d.)	0.6 (1.0)	0.8 (1.2)	0.6 (0.9)	0.9 (1.0)	<0.000
FEV_1_/FVC post-proportion predicted	*n* (%)	57.3 (10.7)	54.2 (11.4)	55.9 (10.6)	55.7 (11.1)	<0.000
FEV_1_ post-proportion predicted	*n* (%)	72.1 (17.4)	66.6 (17.8)	69.7 (19.2)	67.2 (18.2)	<0.000
						
*Smoking*						
Current smoker	*n* (%)	316 (37.6)	194 (58.3)	71 (52.2)	203 (57.5)	<0.000
Never smoker	*n* (%)	29 (3.5)	7 (2.1)	3 (2.2)	16 (4.5)	
Quit ⩾12 months ago	*n* (%)	493 (58.7)	131 (39.3)	62 (45.6)	134 (38.0)	
						
*Clinical COPD Questionnaire(CCQ)*						
Subscale functional status	Mean (s.d.)	0.6 (0.0)	0.9 (0.0)	0.9 (0.1)	1.0 (0.0)	<0.000
Subscale mental status	Mean (s.d.)	0.1 (0.0)	0.2 (0.0)	0.3 (0.1)	0.5 (0.0)	<0.000
Subscale symptom status	Mean (s.d.)	1.3 (0.0)	1.8 (0.0)	1.8 (0.1)	2.2 (0.0)	<0.000
Total score	Mean (s.d.)	0.8 (0.0)	1.1 (0.0)	1.1 (0.0)	1.4 (0.0)	<0.000
						
*Symptoms*						
Sputum^a^	Mean (s.d.)	1.1 (0.0)	1.9 (0.1)	1.7 (0.1)	2.5 (0.1)	<0.000
Wheezing^b^	Mean (s.d.)	0.8 (0.0)	1.3 (0.1)	1.3 (0.1)	1.9 (0.1)	<0.000
Exacerbations^c^	Mean (s.d.)	0.6 (1.0)	0.8 (1.2)	0.6 (0.9)	0.9 (1.0)	<0.000
						
*GOLD Stage*						
1	*n* (%)	299 (35.6)	50 (36.8)	50 (36.8)	121 (34.3)	<0.000
2	*n* (%)	470 (56.0)	68 (50.0)	68 (50.0)	189 (53.5)	
3	*n* (%)	68 (8.1)	17 (12.5)	17 (12.5)	41 (11.6)	
4	*n* (%)	3 (0.4)	1 (0.7)	1 (0.7)	2 (0.6)	
A	*n* (%)	458 (54.5)	84 (25.2)	36 (26.5)	52 (14.7)	<0.000
B	*n* (%)	210 (25.0)	130 (39.0)	62 (45.6)	182 (51.6)	
C	*n* (%)	104 (12.4)	40 (12.0)	14 (10.3)	17 (4.8)	
D	*n* (%)	67 (8.0)	79 (23.7)	24 (17.6)	102 (28.9)	
						
*Medication*						
No medication	*n* (%)	387 (42.6)	164 (36.9)	80 (48.8)	287 (40.1)	0.06
Short-acting bronchodilators	*n* (%)	90 (9.9)	77 (17.3)	24 (14.6)	145 (20.3)	<0.000
Inhaled corticosteroids	*n* (%)	68 (7.5)	39 (8.8)	11 (6.7)	52 (7.3)	NS
Leukotriene antagonists	*n* (%)	3 (0.3)	1 (0.2)	0 (0.0)	1 (0.1)	NS
Short-acting bronchodilator and anticholinergics	*n* (%)	2 (0.2)	0 (0.0)	0 (0.0)	0 (0.0)	NS
Long-acting bronchodilators	*n* (%)	26 (2.9)	25 (5.6)	4 (2.4)	30 (4.2)	0.074
Combination inhaled corticosteroids and long-acting bronchodilators	*n* (%)	277 (30.5)	137 (30.9)	50 (30.5)	206 (28.8)	NS
Long-acting muscarinic antagonists	*n* (%)	223 (24.6)	97 (21.8)	23 (14.0)	144 (20.1)	0.012
Short-acting muscarinic antagonists	*n* (%)	67 (7.4)	32(7.2)	12(7.3)	1 (0.1)	NS
Oral corticosteroids	*n* (%)	0 (0.0)	2 (0.5)	0 (0.0)	57 (8.0)	NS

The total number of patients in this table is 2,231, as 38 patients did not fill in the ACQ.

Abbreviations: COPD, chronic obstructive pulmonary disease; FEV_1_, forced expiratory volume in 1 s; FVC, forced vital capacity; GOLD, Global initiative for chronic obstructive disease; NS, not significant.

a CCQ question 6.

b ACQ question 5.

c Defined as having used antibiotics or oral corticosteroids in the past 12 months for respiratory complaints.

**Table 4:** Regression analyses with three different dependent variables, having an exacerbation, total CCQ score, FEV_1_ predicted, 10–17 months after baseline (*n*=346)

*Predictor*	*Risk factor*	*OR*	*95% CI*	*P value*
*Dependent variable: having an exacerbation, 10–17 months after baseline visit (*n*=346)*				
Baseline exacerbation	No	1		
	Yes	4.13	2.45-6.95	<0.000
Morning symptoms (ACQ question 2)	No (ACQ=0)	1		
	Moderate (ACQ:1–3)	1.24	0.60-2.56	NS
	Severe (ACQ*⩾*4)	1.58	0.76-3.28	NS
Night symptoms (ACQ question 1)	No (ACQ=0)	1		
	Moderate (ACQ:1–3)	1.94	1.02-3.69	0.044
	Severe (ACQ*⩾*4)	1.27	0.52-3.09	NS
				
*Dependent variable: total CCQ* a *score⩾1, 10–17 months after baseline visit (n=345)*				
Baseline total CCQ score	Low symptomatic (CCQ<1)	1		
	High symptomatic (CCQ*⩾*1)	6.48	3.66-11.46	<0.000
Morning symptoms (ACQ question 2)	No (ACQ=0)	1		
	Moderate (ACQ:1–3)	2.75	1.33-5.70	0.006
	Severe (ACQ*⩾*4)	12.22	4.76-31.39	<0.000
Night symptoms (ACQ question 1)	No (ACQ=0)	1		
	Moderate (ACQ:1–3)	1.99	1.01-3.91	0.047
	Severe (ACQ*⩾*4)	2.63	0.81-8.56	NS

*Predictor*	*B*	*s.e.*	t	P *value*
*Dependent variable: FEV*_*1*_ *% predicted, 10–17 months after baseline visit (*n*=345)*				
Constant	7.45	2.44	3.06	0.002
Baseline FEV_1_% predicted	0.90	0.03	28.76	<0.000
Morning symptoms (ACQ question 2)	−0.01	0.63	−0.01	NS
Night symptoms (ACQ question 1)	−0.75	0.52	−1.43	NS

Abbreviations: ACQ, Asthma Control Questionnaire; CI, confidence interval; COPD, chronic obstructive pulmonary disease; FEV_1_, forced expiratory volume in 1 s; CCQ, COPD Clinical Questionnaire; NS, not significant; OR, odds ratio.

a Clinical COPD Questionnaire.

## Notes

IT, NC and JK are associate editors of npj Primary Care Respiratory Medicine, but were not involved in the editorial review of, nor the decision to publish this article. The other authors declare no conflict of interest.
